# Pulmonary focal fibrosis associated with microscopic arterio-venous fistula manifesting as focal ground-glass opacity on thin-section CT

**DOI:** 10.1186/1471-2466-13-3

**Published:** 2013-01-14

**Authors:** Noriko Sudo, Atsushi Nambu, Takana Yamakawa, Masashi Kawamoto, Shozo Fujino, Masato Watanabe, Kunio Mizuguchi, Masao Tago

**Affiliations:** 1 Department of Radiology, Teikyo University Mizonokuchi Hospital, 3-8-3 Mizonokuchi Takatsu-ku, Kawasaki City, Kanagawa, 213-8507, Japan; 2 Department of Clinical Pathology, Teikyo University Mizonokuchi Hospital, 3-8-3 Mizonokuchi Takatsu-ku, Kawasaki City, Kanagawa, 213-8507, Japan; 3 Department of Surgery, Teikyo University Mizonokuchi Hospital, 3-8-3 Mizonokuchi Takatsu-ku, Kawasaki City, Kanagawa, 213-8507, Japan

**Keywords:** Ground-glass opacity, Focal fibrosis, Lung, CT, Arterio-venous fistula

## Abstract

**Background:**

Focal ground-glass opacity (GGO) on thin-section computed tomography (CT) may be seen in atypical adenomatous hyperplasia (AAH), adenocarcinoma in situ that has recently been renamed from bronchioloalveolar carcinoma (BAC) and various benign conditions.

**Case presentation:**

We report a case of pulmonary focal fibrosis associated with microscopic arterio-venous fistula (AVF), which showed a focal area of GGO on thin-section CT. The patient was a 58-year-old woman with a GGO on thin-section CT which had increased in size over the period of 2 years. Slightly dilated vessels and thickened interlobular septa were also noted around the GGO. It was diagnosed preoperatively as adenocarcinoma in situ and a partial lung resection by video-assisted thoracic surgery (VATS) was performed. Pathological examination yielded a diagnosis of focal fibrosis associated with microscopic AVF.

**Conclusion:**

We speculate that the focal fibrosis was produced by a prolonged congestion due to the AVF and that the dilated vessels and thickening of interlobular septa on thin-section CT related to the AVF. Microscopic AVF may be one of the etiologies of focal fibrosis showing focal GGO on thins-section CT. Dilated vessels and thickened interlobular septa around the GGO might offer a clue to the diagnosis of this disease entity. In addition, it should be noted that focal fibrosis may increase in size.

## Background

Recently, widespread use of computed tomography (CT) for lung cancer screening allows us to detect many faint peripheral pulmonary lesions. Radiologists have come to encounter focal ground-glass opacity (GGO) more frequently than before. Persistent focal GGO is most commonly seen in atypical adenomatous hyperplasia (AAH) and adenocarcinoma in situ that has recently been renamed from bronchioloalveolar carcinoma (BAC) in the revised classification [1]. Focal GGO may also be seen in various benign conditions, including focal fibrosis, focal inflammation, or hemorrhage [2,3]. Focal fibrosis accounts for approximately 15% of focal pure GGO [4]. However its etiology is still unknown.

Herein, we present a case of focal fibrosis associated with microscopic arterio-venous fistula (AVF) manifesting as focal GGO mimicking adenocarcinoma in situ on chest CT, which suggested a pathogenesis of focal fibrosis.

## Case presentation

**Figure 1:**
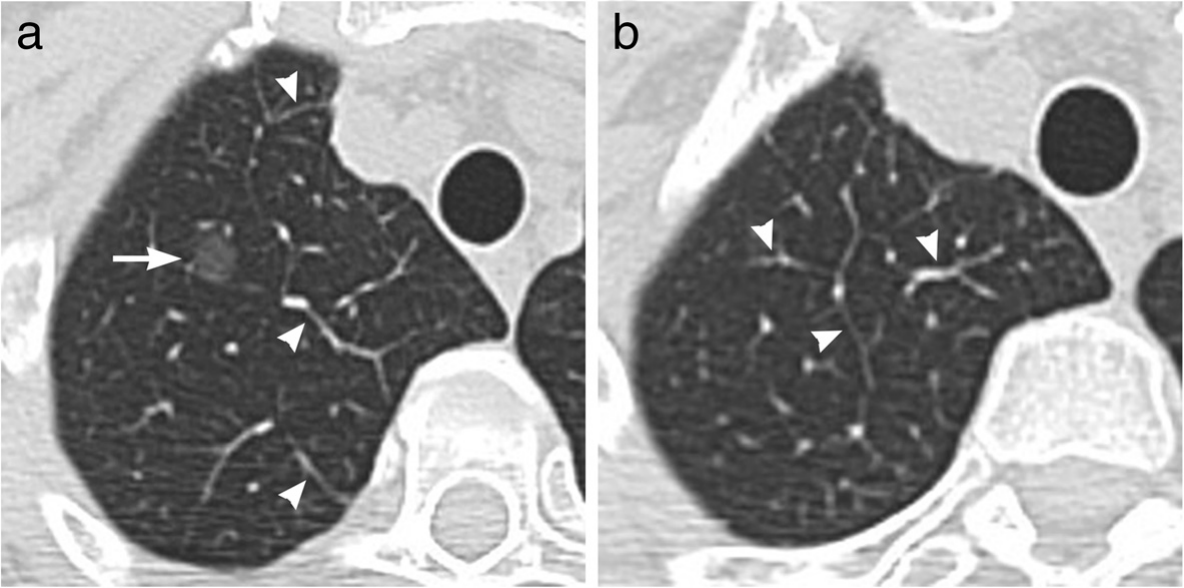
**Chest thin-section CT on admission to our hospital.** (**a**) A thin-section CT at the level of right upper lobe shows a 10-mm round, well-defined GGO nodule (*arrow*). There are slightly dilated veins or thickening of the interlobular septa (*arrow heads*). (**b**) Similar findings are seen also on the slice just above the focal GGO (arrow heads).

A 58-year-old woman was referred to our hospital for detailed evaluation of focal GGO that had been discovered by a screening CT two years before. Thirteen years earlier, the patient had undergone a partial left lung resection for pulmonary hamartoma. Biochemical tests and tumor markers for lung cancer on admission were within the normal ranges. Her physical condition was good with no clinical symptoms. She had never smoked. Chest thin-section CT scan showed a 10-mm pure GGO with a well-defined and smooth margin in the right upper lobe (Figure 1a). In addition, slightly dilated veins and thickening of interlobular septa were seen around the focal GGO (Figure 1a, b).

**Figure 2:**
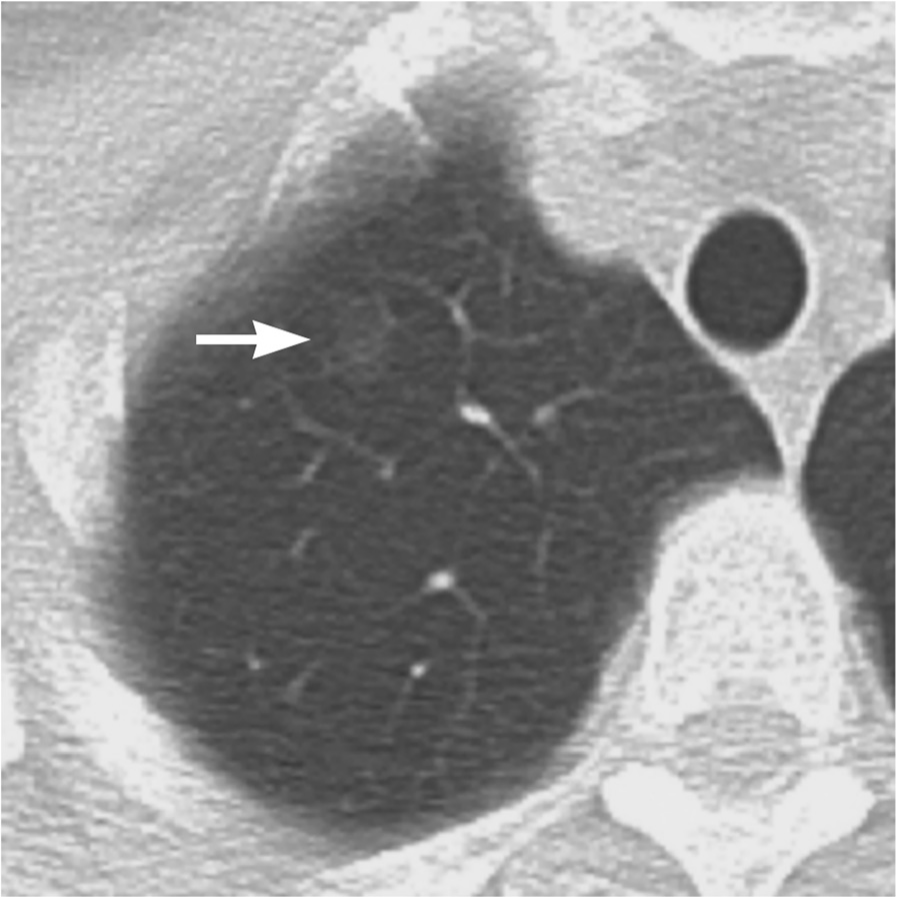
**Screening chest CT at 2 years before CT examination at our hospital.** A 7mm slice thickness CT shows a faint pure GGO in the right upper lobe (*arrow*), which is smaller than that of Figure 1.

**Figure 3:**
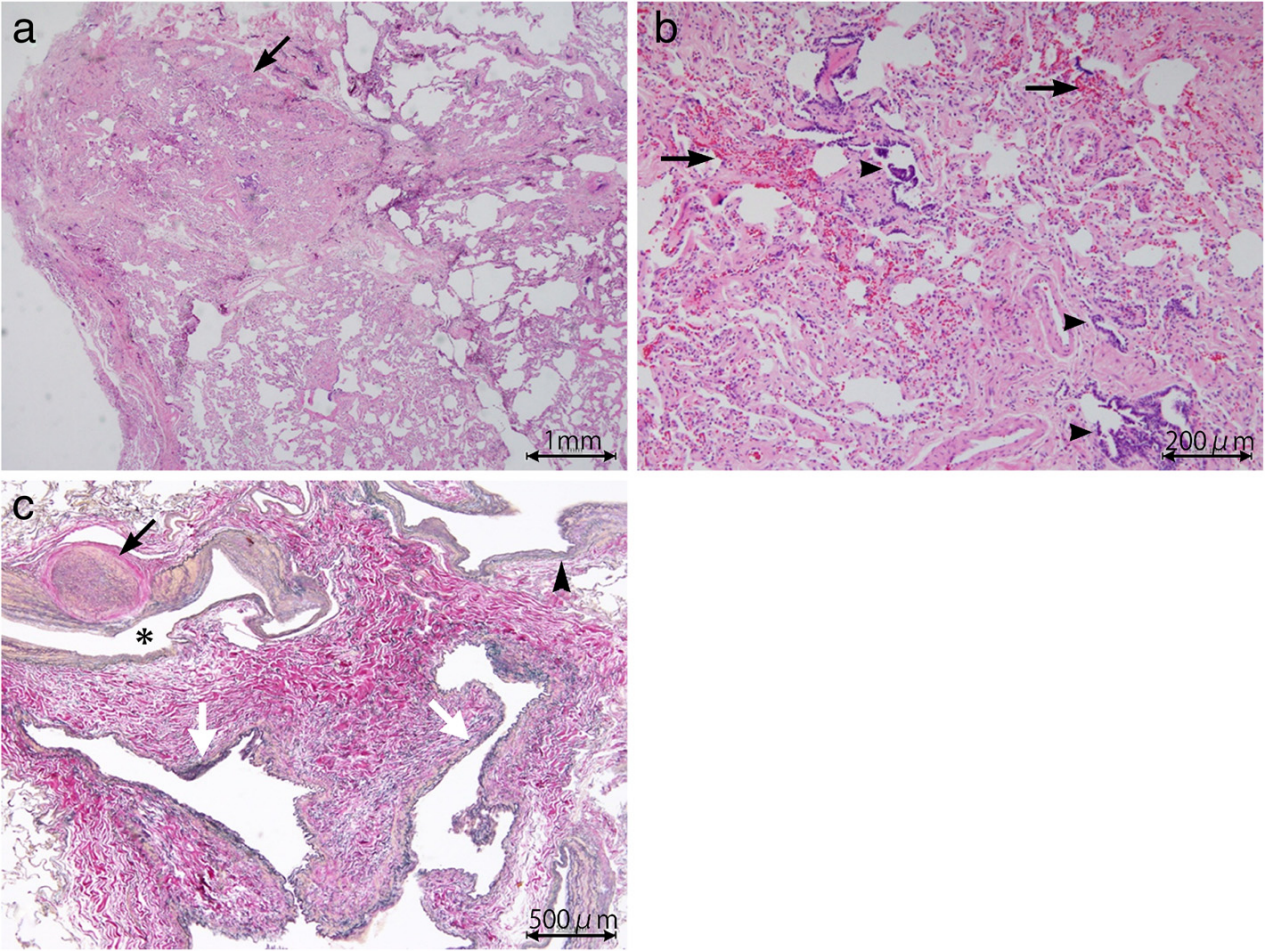
**Histopathology of the resected lung tissue.** (**a**) Photomicrograph (hematoxylin-eosin stain; original magnification, ×2) shows a focal area of alveolar wall thickening with preservation of the intraalveolar airspaces, consistent with the focal GGO on thin-section CT (*arrow*). Partial collapse of this lesion is due to an artifact during the process of specimen production. (**b**) Photomicrograph (hematoxylin-eosin stain; original magnification, ×10) shows alveolar wall thickening with fibrosis, and congestion(*arrows*). Alveolar bronchiolizations are also seen (*arrowheads*). (**c**) Photomicrograph (elastica Masson stain; original magnification, ×4) near the focal fibrosis shows dilated arterioles (*asterisk*) with markedly thickened walls, one of which have resulted in luminal obliteration (*black arrow*), as well as dilated venules (*white arrows*). A transition from arteriolar wall to venular one is seen, suggesting a direct communication between them (*arrow head*).

The focal GGO was identifiable also on the previous CT for lung cancer screening when viewed retrospectively (Figure 2). It had increased in size for 2 years. The lesion was not identified on chest radiograph. Adenocarcinoma in situ was considered likely due to the presence of an interval increase of the GGO. The lesion was resected by video-assisted thoracic surgery (VATS). It was unidentifiable during the surgery. Histopathological examination of the resected lung tissue revealed no evidence of malignancy but abnormally dilated arterioles, venules and lymph ducts with an area of peripheral focal fibrosis consisting of congestion, fibrotic septal thickening with preservation of the intraalveolar airspaces (Figure 3a–c). This focal fibrosis was considered to correspond to the focal GGO on thin-section CT. The dilated arterioles had thickened walls and were directly communicating with the venules (Figure 3c). These histopathological findings were similar to those of arteriovenous malformation (AVM). However, the present case showed no significant evidence of nidus and it was unknown whether these vascular abnormalities were congenital or not. Therefore, we regarded it as microscopic AVF.

## Discussion

Pure GGO that is persistently (either no change or an increase in diameter for ≧1 month) present on serial thin-section CT scans suggests the possibility of AAH, adenocarcinoma in situ, pulmonary lymphoproliferative disorder, or organizing pneumonia/focal fibrosis [5]. The most common diseases showing persistent focal GGO are AAH and adenocarcinoma in situ. Focal fibrosis is a relatively rare disease entity constituting 15–21% of persistent focal GGO [3,4].

The pathogenesis of focal fibrosis is still not well understood. It may represent a focal tissue response to local lung injury due to infection or radiation, drug, or physical pressure [6]. In our case, microscopic AVF coexisted with the focal fibrosis. We speculate that prolonged localized congestion caused by AVF lead to focal tissue fibrosis. Of note in our case is that the focal fibrosis had increased in size during the time interval between the two CT examinations. As congestion associated with AVF is considered a chronic pathophysiological process, the fibrosis may have gradually extended.

Focal fibrosis has been described as sharply demarcated nodular GGO with a maximal diameter of less than 2 cm on thin-section CT [4]. Solid components which pathologically correspond to compact fibrotic foci or alveolar collapse may be present. These imaging features are shared by both focal fibrosis and AAH/ adenocarcinoma in situ. Therefore, the differentiation between these lesions by CT images is difficult. One possible discriminating feature may be that focal fibrosis does not increase in size over a considerable period of time [6]. However, our case showed an interval growth over a few years.

F-18 fluorodeoxyglucose positron emission tomography (^18^F-FDG PET) has increasingly been used in the evaluation of lung cancer. It is also useful for differentiating between malignant and benign nodules. However, as for focal GGO, it has been shown that ^18^F-FDG PET has limited value in the evaluation of focal GGOs for determining nodule malignancy and staging in comparison with solid nodules. Chun et al. [7] reported that in pure GGOs, both inflammation and malignancy showed an SUV less than 1.0 and did not show a statistically significant difference. Therefore, ^18^F-FDG PET is considered to contribute little to the differentiation between adenocarcinoma in situ and focal fibrosis.

A well-known imaging feature of pulmonary AVF is dilatation of the feeding artery and draining vein with a nodular area where these vessels join. We couldn’t see such a nodular area in our case probably because the AVF was microscopic and thus was too small to see on CT. However, slightly dilated veins and thickened interlobular septa were demonstrated around the focal GGO on thin-section CT. We think that these findings related to the AVF and may be a clue to the differentiation between this disease entity and AAH/adenocarcinoma in situ.

## Conclusions

In summary, we presented a rare case of focal fibrosis associated with microscopic AVF that showed focal GGO on thin-section CT. To our knowledge, this is the first report of this disease entity. Although the CT findings are similar to those of AAH/adenocarcinoma in situ, dilated vessels and thickened interlobular septa around the GGO might offer a clue to the differential diagnosis. Additionally, it should be noted that focal fibrosis may increase in size.

## Consent

Written informed consent was obtained from the patient for publication of this Case report and any accompanying images. A copy of the written consent is available for review by the Editor of this journal.

## Abbreviations

AVF: Arterio-venous fistula; GGO: Ground-glass opacity; CT: Computed tomography; BAC: Bronchioalveolar carcinoma; VATS: Video-assisted thoracic surgery; AAH: Adenomatous hyperplasia; AVM: Arteriovenous malformation; ^18^F-FDG PET: F-18 fluorodeoxyglucose positron emission tomography.

## Competing interests

We have no competing interests.

## Authors’ contributions

NS drafted the initial manuscript and modified it in reference to the other authors’ opinion. AN, TY and MT edited the manuscript. MK, SF, MW and KM were involved in diagnostics and treatment of the patient and provided advice to me in each of their expertise areas. All authors read and approved the final manuscript.

## Pre-publication history

The pre-publication history for this paper can be accessed here:

http://www.biomedcentral.com/1471-2466/13/3/prepub
